# Supplementary material for: Physicochemical Characterization and Antibacterial Properties of Carbon Dots from Two Mediterranean Olive Solid Waste Cultivars
Source: Nanomaterials (Basel). 2022 Mar 7;12(5):885. doi: 10.3390/nano12050885 (PMC8912711; doi:10.3390/nano12050885)
Supplement: Supplementary file 1 [file nanomaterials-12-00885-s001.zip › nanomaterials-1615742-supplementary.pdf]

nanomaterials

Supplementary Material

Physicochemical Characterization and Antibacterial Properties of Carbon Dots From Two Mediterranean Olive Solid Waste Cultivars

Giuseppe Nocito [1,†], Emanuele Luigi Sciuto [1,†], Domenico Franco [1], Francesco Nastasi [1], Luca Pulvirenti [2], Salvatore Petralia [3], Corrado Spinella [4], Giovanna Calabrese [1,*], Salvatore Guglielmino [1] and Sabrina Conoci [1,4,5,6 *]

1. Department of Chemical, Biological, Pharmaceutical and Environmental Sciences, University of Messina, Viale Ferdinando Stagno d'Alcontres, 31, 98168 Messina, Italy; sabrina.conoci@unime.it (S.C.); gnocito@unime.it (G.N.); emanueleluigi.sciuto@unime.it (E.L.S.); dfranco@unime.it (D.F.); fnastasi@unime.it (F.N.); gcalabrese@unime.it (G.C.); salvatore.guglielmino@unime.it (S.G.)
2. Department of Chemical Science, University of Catania, Viale A. Doria, 6, 95125 Catania, Italy; luca.pulvirenti@phd.unict.it
3. Department of Drug Science and Health, University of Catania, Viale A. Doria, 6, 95125 Catania, Italy; salvatore.petralia@unict.it
4. Istituto per la Microelettronica e Microsistemi, Consiglio Nazionale delle Ricerche (CNR-IMM) Zona Industriale, VIII Strada 5, 95121 Catania, Italy; corrado.spinella@imm.cnr.it
5. Department of Chemistry ''Giacomo Ciamician'', University of Bologna, Via Selmi 2, 40126 Bologna, Italy
6. LabSense Beyond Nano – URT Department of Physic –CNR Viale Ferdinando Stagno d'Alcontres, 31, 98168 Messina, Italy

* Correspondence: sabrina.conoci@unime.it (S.C.) and gcalabrese@unime.it (G.C.)

† equal contribution

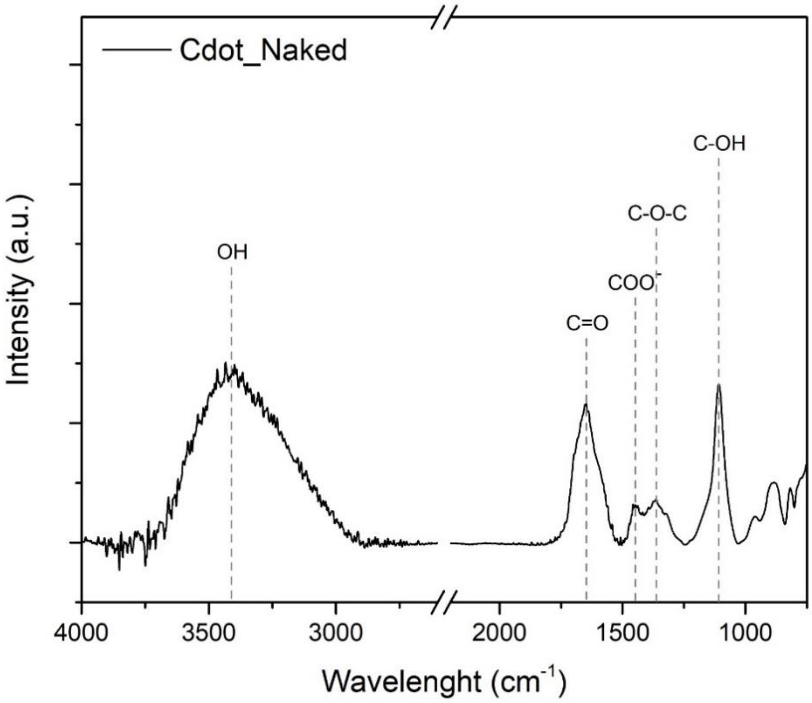

Figure S1. FTIR Spectra CDs_C.

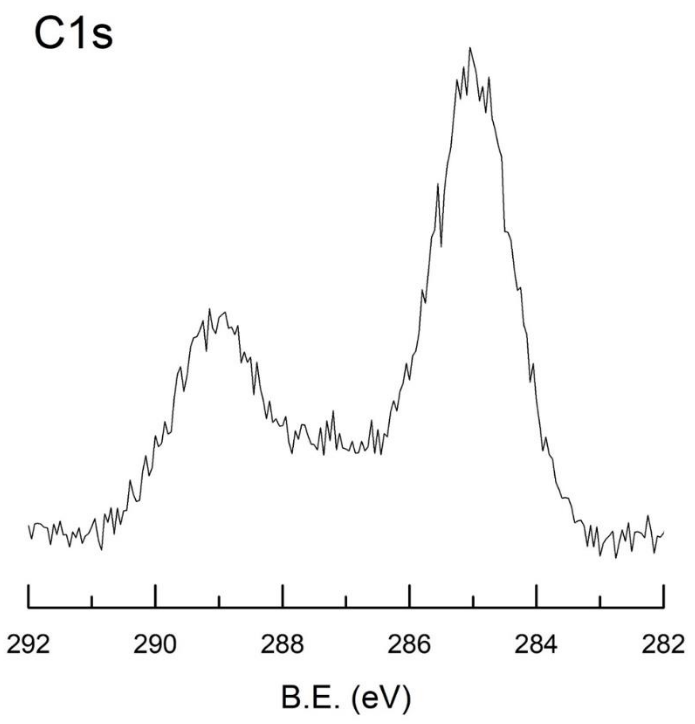

Figure S2. XPS Spectra CDs_C.

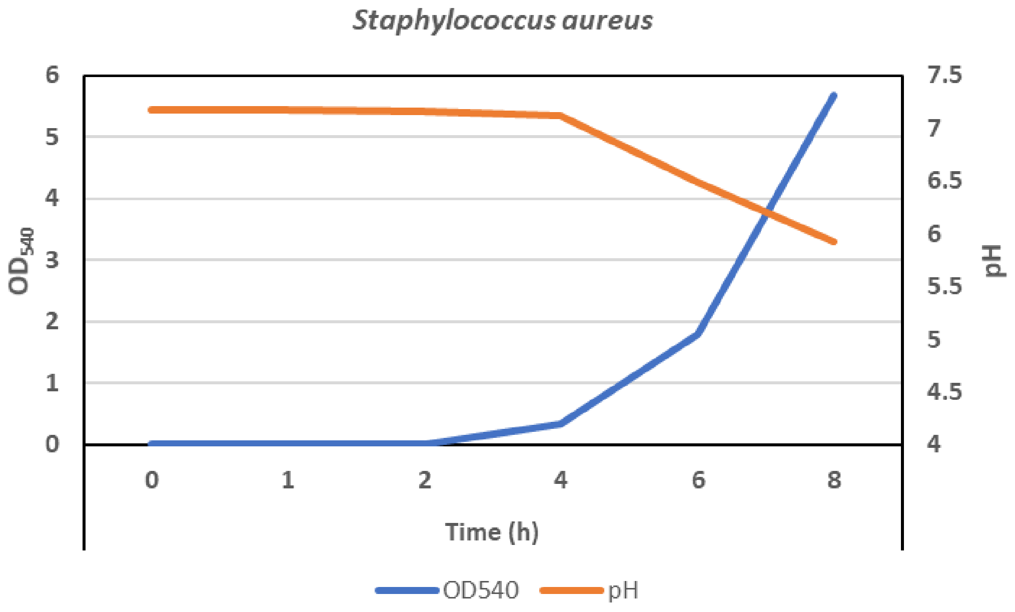

Figure S3. pH changes during *S. aureus* bacterial growth (OD_{540}) (0–8hrs).
